# Acute exposure to organophosphorus pesticide metabolites compromises buffalo sperm function and impairs fertility

**DOI:** 10.1038/s41598-023-35541-6

**Published:** 2023-06-05

**Authors:** Shivani Chhillar, Vipul Batra, Arumugam Kumaresan, Rakesh Kumar, Ankit Pal, Tirtha Kumar Datta

**Affiliations:** 1 Animal Genomics Lab., Animal Biotechnology Centre, ICAR-NDRI, National Dairy Research Institute, Karnal, India; 2 School of Medicine, Division of Child Health, Obstetrics and Gynecology, University of Nottingham, Nottingham, England; 3 Theriogenelogy Lab., SRS of National Dairy Research Institute, Bengaluru, India; 4 ICAR-Central Institute for Research on Buffaloes, Hisar, India

**Keywords:** Cellular imaging, Mechanism of action

## Abstract

Agrichemicals such as organophosphorus pesticides’ metabolites (OPPMs) are more hazardous and pervasive than their parent pesticides. Parental germline exposure to such xenobiotics leads to an elevated susceptibility towards reproductive failures e.g. sub- or in-fertility. This study sought to examine the effects of low-dose, acute OPPM exposure on mammalian sperm function using buffalo as the model organism. The buffalo spermatozoa were briefly (2 h) exposed to metabolites of the three most prevalent organophosphorus pesticides (OPPs) viz. Omethoate (from Dimethoate), paraoxon-methyl (from methyl/ethyl parathion) and 3, 5, 6-trichloro-2-pyridinol (from chlorpyrifos). Exposure to OPPMs resulted in compromised structural and functional integrity (dose-dependent) of the buffalo spermatozoa typified by elevated membrane damage, increased lipid peroxidation, precocious capacitation and tyrosine phosphorylation, perturbed mitochondrial activity and function and (P < 0.05). This led to a decline in the in vitro fertilizing ability (P < 0.01) of the exposed spermatozoa, as indicated by reduced cleavage and blastocyst formation rates. Preliminary data indicate that acute exposure to OPPMs, akin to their parent pesticides, induces biomolecular and physiological changes in spermatozoa that compromise their health and function ultimately affecting their fertility. This is the first study demonstrating the in vitro spermatotoxic effects of multiple OPPMs on male gamete functional integrity.

## Introduction

OPPs are amongst the most widely used synthetic pesticides around the globe. Despite being banned or restricted in many developed nations, their exposure is still prevalent, particularly in developing economies across the globe^1–3^. Consequently, a large fraction of the human and animal population is exposed to such pesticides rendering these living organisms vulnerable to various reproductive hazards^4–7^. Most pesticides including the OPPs contain at least one agent/metabolite that affects any of the reproductive or developmental endpoints in multiple mammalian species including humans^8–10^. The OPPs and their metabolites (OPPMs) are known mammalian male reproductive toxicants that damage the DNA of the spermatozoa, alter testicular-somatic cells’ function and adversely affect the semen quality (genotoxic and teratogenic effects)^11–13^.

The metabolites produced after the biotransformation of pesticides are considered more hazardous than their parent pesticides and they often persist, pervade the environment, and interact with various organisms for decades after exposure leading to widespread body burdens^14–17^. For example, the OPP, Dimethoate, *O*, *O*-Dimethyl *S*-[2-(methylamino)-2-oxoethyl] phosphorodithioate, a systemic and contact OPP insecticide and its metabolite, Omethoate, O, O-Dimethyl S-[2-(methylamino)-2-oxoethyl] phosphorothioate reportedly persist in the reproductive organs of mice and rats for weeks after exposure^18–20^. Omethoate is the toxic metabolite of dimethoate that has been implicated in pesticide toxicity in insects and mammals^21,22^. Epidemiological data and experimental studies indicate a correlation between exposure to OPPs and perturbed sperm functional parameters (SFPs) and a decline in fertility^23–25^. Spermatozoa are particularly vulnerable to the adverse effects of various agrichemical exposures and insults. The near lack of apoptotic mechanisms; repair, proofreading and protective enzymes; exceptionally high surface area/volume ratio (> 50:1) and a considerable proportion of unsaturated fatty acids (PUFAs) in membrane render them comparatively more susceptible to environmental insults vis-à-vis the female gamete and other somatic cells^26–29^. Besides, the male-specific traits e.g., the higher abundance of androgen receptors which can interact with several pesticides, altered testicular pathology, a decline in sperm count and reduction in sperm motility are also implicated in the elevated severity observed in male reproductive function^30–34^**.**

Notably, under the Indian grazing systems the livestock animals are reared as per the semi-intensive farming paradigm wherein the animals (e.g. buffaloes) are let out for grazing in open fields rather than being stall-fed^35–39^. This greatly increases their chances of exposure to agrichemicals such as OPPs. We chose buffalo as a model for this study because it is a primary dairy animal in South and Middle Eastern Asian countries^40^. A crucial milch and meat species, more people depend on buffalo for their livelihood than on any other livestock animal^40^. Nevertheless, it suffers from numerous reproductive constraints e.g., low conception rates (sub-fecundity) despite producing morphologically normal spermatozoa (idiopathic causes).

The anti-androgenic effects and the alterations in the reproductive enzyme pathways upon acute or chronic OPP exposure are known to negatively influence the mammalian spermatozoa quality and function apart from causing pernicious effects in the offspring^41–44^. Consequently, interest in defined, low-dose, acute or chronic pesticide exposure on the male reproductive system has recently increased apparently because of the lack of a consented definition of the chronic or low-level exposures in many nations, which makes it difficult to decide the class of the exposure^23,44–46^. Nonetheless, only a limited number of studies have investigated the effect of acute, in vitro OPPM exposure on livestock sperm health, function and fertility. Against this background, this work aimed to investigate the effects of low-dose, acute exposure to three OPP metabolites viz. omethoate (from dimethoate), paraoxon-methyl (from methyl parathion) and 3, 5, 6-trichloro-2-pyridinol or TCPy (from chlorpyrifos) on mammalian sperm function following exposure for 2 h at 38 °C. We used the buffalo sperm as the model and tested a continuum of doses ranging from 0.5 to 20 µM, based on previous in vitro OPP reproductive toxicology studies since the mammalian spermatozoa from larger animals reportedly serve as the best potential alternative to the use of live mammalian model organisms^47–49^.

## Results

The mean representative motility of the control, vehicle (DMSO) control and the OPPM exposed spermatozoa are shown in Supplementary Fig. 1. No changes in the motility of the buffalo spermatozoa upon exposure to DMSO or at low doses (< 5 μM) were observed (Supplementary Fig. 1).

### Sperm function parameters (SFPs)

#### Membrane integrity assessment

**Figure 1:**
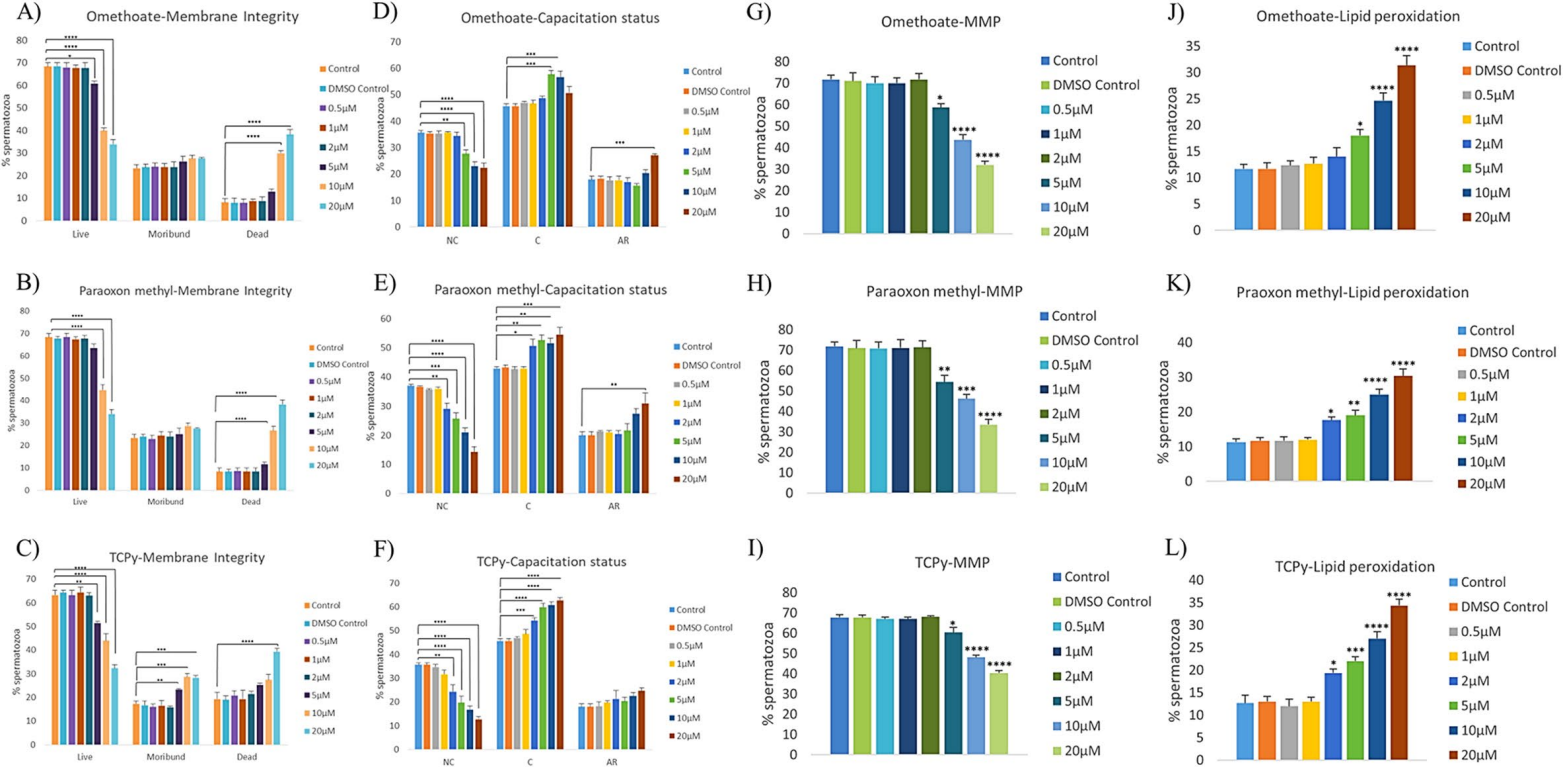
Sperm Functional Parameters. The buffalo spermatozoa were exposed to 0.5, 1, 2, 5, 10 and 20 μM of Omethoate, Paraoxon methyl, TCPy for the assessment of membrane integrity (**A**–**C**), capacitation status (**D**–**F**), mitochondrial membrane potential (**G**–**I**) and lipid peroxidation (**J**–**L**) using fluorescent staining. CFDA with PI (see text) was used to assess the membrane integrity of the spermatozoa that were categorized as live, dead or moribund. CTC (see text) was used to categorize the spermatozoa as non-capacitated (NC), capacitated (C), or acrosome-reacted (AR). JC-1 (see text) was used to calculate the percentage of spermatozoa with high and low mitochondrial membrane potential while BODIPY (see text) was used to distinguish the spermatozoa with high and low lipid peroxidation (LPO).

Membrane integrity is a crucial morphological/structural criterion for a spermatozoon to traverse the FRT and fertilize the oocyte. The CFDA-PI dual staining discerned three spermatozoa populations viz. live, dead and moribund (dying sperm, ROS producers) spermatozoa based on their fluorescence patterns which are indicative of the functional or compromised membrane integrity of mammalian sperm (Supplementary Fig. 2A). The structural integrity of the buffalo spermatozoa plasma membrane appeared to be compromised in a dose-dependent manner upon incubation with omethoate (Fig. 1A), paraoxon methyl (Fig. 1B), and TCPy (Fig. 1C).

#### Capacitation status

Capacitation is an imperative process that a spermatozoon must go through before attaining the ability to fertilize an egg. Since the membrane dynamics undergo dramatic changes during capacitation, the spermatozoa have a limited shelf life thereafter and as expected, premature capacitation would negatively affect fertility. Chlortetracycline (CTC), which is used to detect Ca^2+^-related changes in the intracellular calcium re-distribution in the sperm head, particularly during capacitation, discerned three distinct fluorescent patterns. These were classified as non-capacitated (NC), capacitated (C) and acrosome-reacted (AR) in the control group and the OPPM exposed spermatozoa (Fig. 1D–F and Supplementary Fig. 2B). The exposure to OPPMs induced precocious capacitation in buffalo spermatozoa which increased linearly with concentration upon incubation with omethoate (Fig. 1D), paraoxon methyl (Fig. 1E), and TCPy (Fig. 1F).

#### Mitochondria membrane potential

The mitochondria membrane potential (MMP) is an indicator of health since the mitochondrion is an important source of ATP. The JC-1 dye was used for the assessment of buffalo spermatozoal MMP post-exposure to OPPMs. It renders the mitochondria with high MMP as fluorescing bright green whereas the mitochondria with low membrane potential produced a dull fluorescence after labelling (Supplementary Fig. 2C). The MMP of buffalo spermatozoa diminished significantly upon incubation with OPPMs. Consequently, a dose-dependent decline in the percentage of spermatozoa with a high mitochondrial membrane potential upon incubation with omethoate (Fig. 1G), paraoxon methyl (Fig. 1H), and TCPy (Fig. 1I) was observed.

#### Lipid peroxidation

Lipid peroxidation has been implicated in the aetiology of defective spermatozoa (with high ROS) function in many mammalian species. The fluorophore, BODIPY ((4,4-Difluoro-1,3,5,7,8-Pentamethyl-4-Bora-3a,4a-Diaza-s-Indacene)) can incorporate into the spermatozoa and undergo a spectral emission shift upon interacting with reactive oxygen metabolites, indicating their oxidative stress. The incubation of buffalo spermatozoa with omethoate (Fig. 1J), paraoxon methyl (Fig. 1K), and TCPy (Fig. 1L) induced lipid peroxidation especially in the mid-piece as observed by BODIPY staining (Supplementary Fig. 2D). However, it was less evident in the sperm head or the rest of the sperm tail (Supplementary Fig. 2D).

### Protein tyrosine phosphorylation

**Figure 2:**
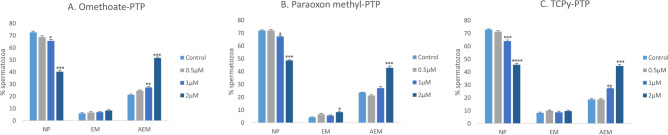
Protein tyrosine phosphorylation. Three major fluorescent patterns viz. non-fluorescent spermatozoa i.e. non-phosphorylated (NP), sperm bearing signal at equatorial region and mid-piece (EM) and spermatozoa with a signal at the acrosomal region, equatorial region and mid-piece (AEM).were observed during immunocytochemistry using a monoclonal anti-phosphotyrosine antibody (P1869; Sigma). The buffalo spermatozoa were exposed to 0.5, 1, and 2 μM of OPPMs viz. Omethoate (**A**), Paraoxon methyl (**B**), and TCPy (**C**) for 2 h.

Exposure to OPPs/OPPMs may cause multiple asymptomatic effects at comparatively lower exposures rather than overt signs and symptoms^50^. We report similar subclinical toxic effects, particularly at doses ≤ 5 µM. Although these doses did not affect the sperm morphology, mass motility or any functional parameters (except capacitation), nonetheless, low doses (1 µM) appeared to negatively affect the phosphorylation status of the protein tyrosine residues present on the cell surface (Fig. 2 and Supplementary Fig. 3). Therefore, only the OPPM concentrations ≤ 5 µM were used in the subsequent experiments. A higher abundance of non-phosphorylated (NP) spermatozoa vis-à-vis the phosphorylated spermatozoa (EM and AEM patterns) was observed in the control and the OPPM treated samples (Fig. 2A–C and Supplementary Fig. 3). Notably, the localization of phosphorylated proteins on the sperm mid-piece always coincided with that of the equatorial region. The incubation with OPPMs appeared to induce the phosphorylation of tyrosine residues (a conserved feature of mammalian sperm capacitation) in the sperm proteins^51^ thus causing a reduction in the fraction of NP spermatozoa (Fig. 2).

### Expression dynamics of metabolic genes and oxidative stress-related genes

**Figure 3:**
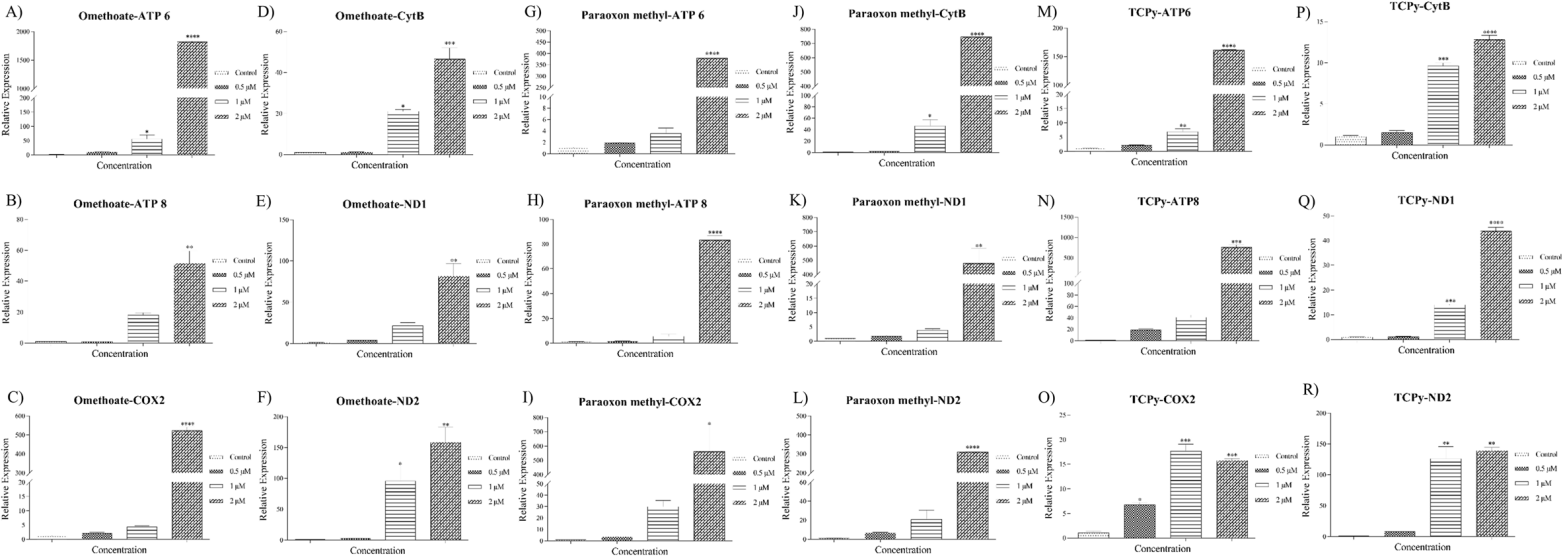
Pattern of expression of metabolic and oxidative stress-related genes. Relative expression profiles of the mitochondrial metabolic genes viz. ATP6, ATP8, COX-2, CytB, ND-1 and ND-2 for the control group and the buffalo spermatozoa exposed to 0.5, 1, and 2 μM of Omethoate (**A**–**F**), Paraoxon methyl (**G**–**L**), and TCPy (**M**–**R**) were generated using RT-qPCR. The expression values are normalized to GAPDH and β-actin, and the error bars represent the standard error of the mean (SEM).

The relative expression profiles of the selected panel of metabolic genes (ATP 6, ATP 8, COX 2, CYT B, ND1 and ND2) from the mitochondrial genome were generated using RT-qPCR in the spermatozoa treated with OPPMs (Fig. 3). The exposure to OPPMs viz. Omethoate (Fig. 3A–F), paraoxon methyl (Fig. 3G–L), and TCPy (Fig. 3M–R) induced the expression of mitochondrial metabolic genes.

### Computer-assisted sperm analysis (CASA)

**Figure 4:**
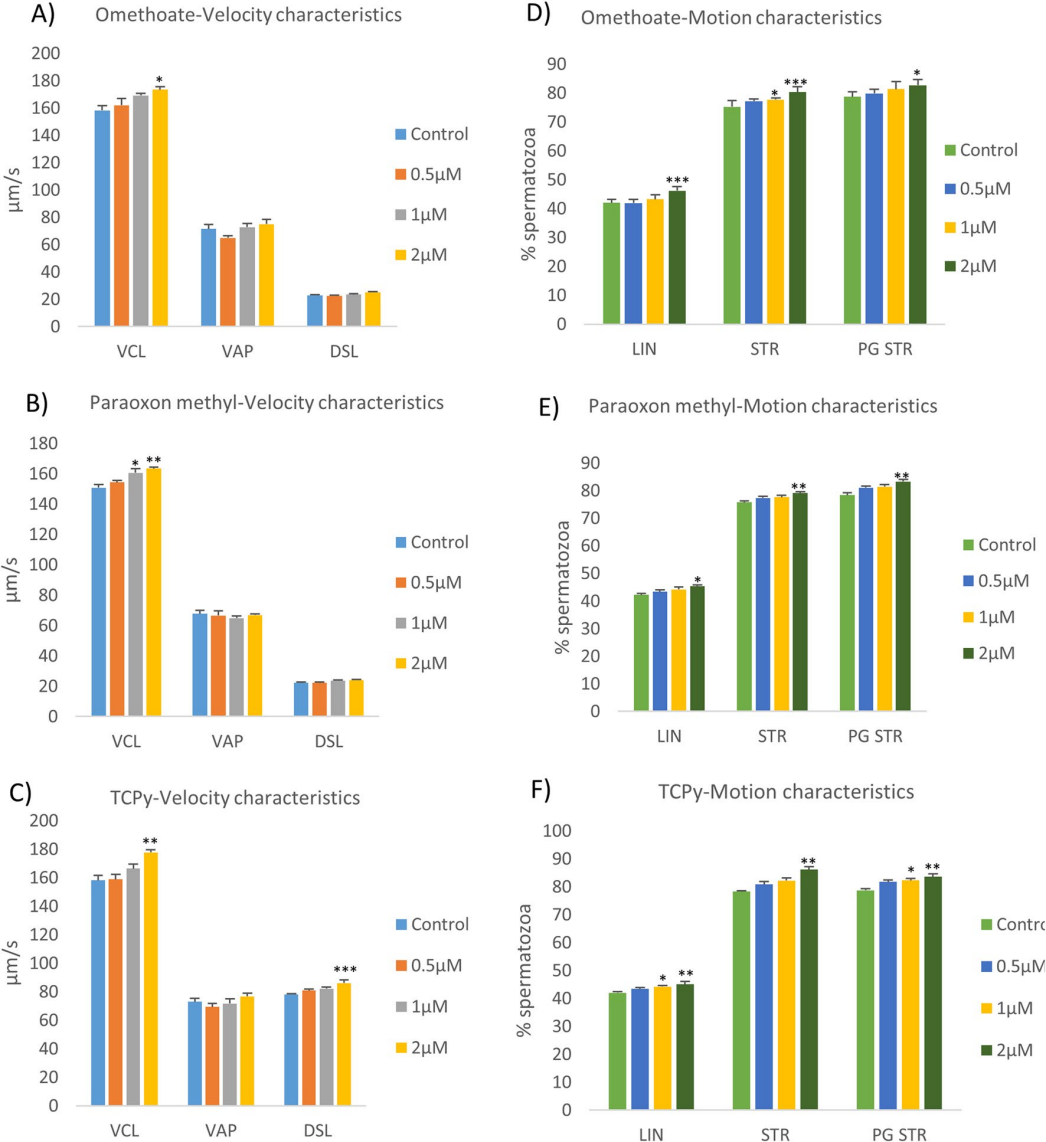
Kinematics of buffalo spermatozoa. The velocity characteristics viz. curvilinear velocity (VCL), average path velocity (VAP) and (DSL) (**A**–**C**) and the motion characteristics viz. the percentage of linearity (LIN), the straightness coefficient (STR) and progressive STR (PGSTR) (**D**–**F**) were evaluated in the buffalo spermatozoa exposed to 0.5, 1, and 2 μM of Omethoate (**A**, **D**), Paraoxon methyl (**B**, **E**), TCPy (**C**, **F**) by Computer-assisted sperm analyzer (IVOS12.1, Hamilton-Thorne Biosciences, Beverly, MA, USA).

The buffalo sperm kinematic parameters were found to be significantly perturbed upon exposure to OPPMs (Fig. 4 and Supplementary Fig. 4). It is worth mentioning that although the lower doses of OPPMs didn’t affect the motility of the buffalo spermatozoa per se, their motion and velocity characteristics were however, altered upon exposure to Omethoate (Fig. 4A,D),Pparaoxon methyl (Fig. 4B,E), and TCPy (Fig. 4C,F). None of the OPPMs affected the lateral head displacement (Supplementary Fig. 4A–C) while only the Omethoate exposure significantly affected (P < 0.01) the beat cross frequency of the buffalo spermatozoa at 2 μM concentration (Supplementary Fig. 4D–F).

### In vitro fertilization (IVF)

**Figure 5:**
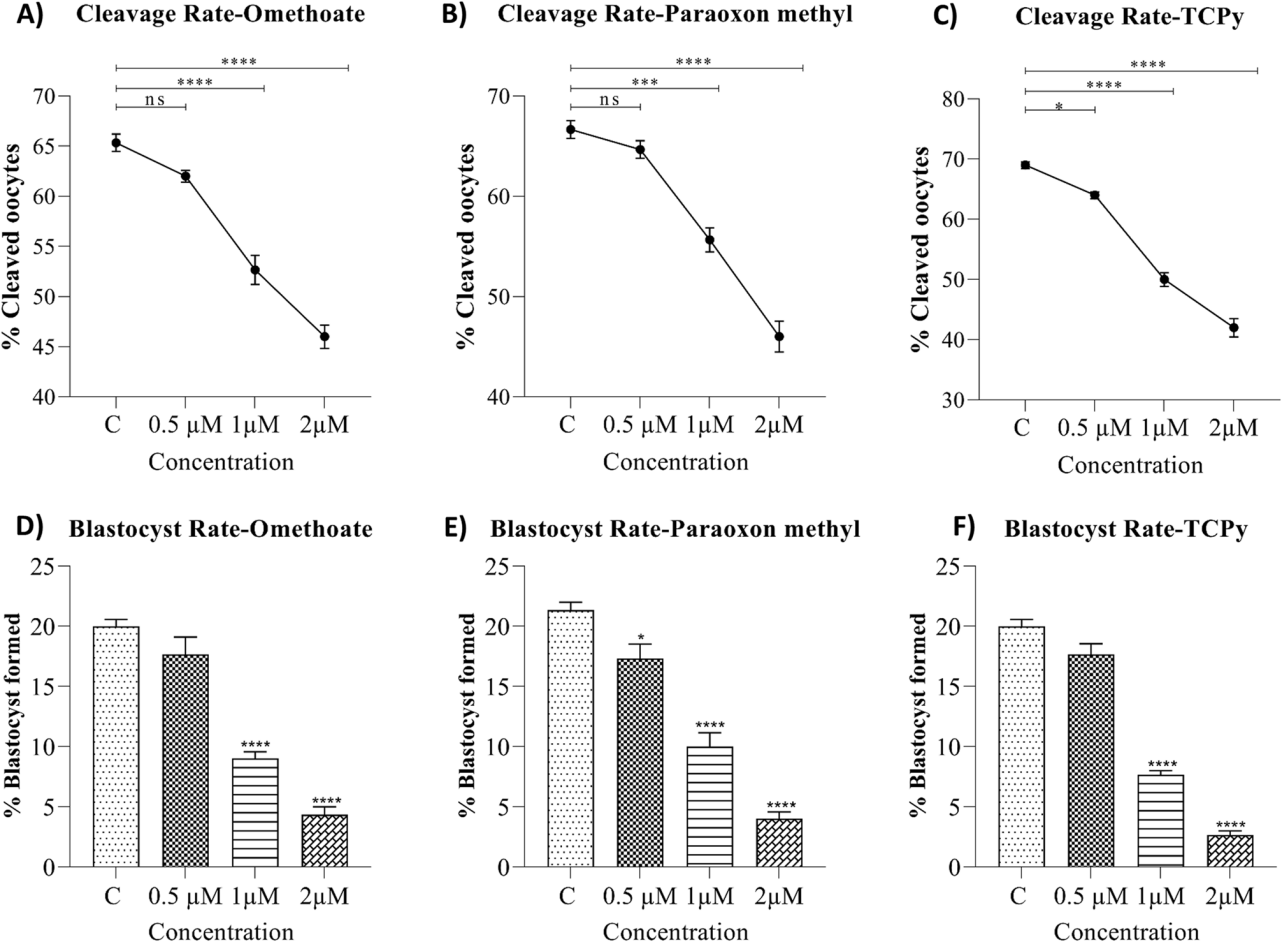
In vitro fertilization. The mean ± SEM for cleavage rate (**A**–**C**), and blastocyst formation rates (**D**–**F**) in the control group and buffalo spermatozoa exposed to 0.5, 1, and 2 μM of OPPM viz. Omethoate (**A**, **D**), Paraoxon methyl (**B**, **E**) and TCPy (**C**, **F**).

The incubation of the semen sample with pesticide metabolites hindered the fertilization in a dose-dependent manner (Fig. 5). The rate of oocyte cleavage (Fig. 5A–C) and formation of the blastocyst (Fig. 5D,E) declined significantly upon exposure to Omethoate (Fig. 5A,D), Paraoxon methyl (Fig. 5B,E), and TCPy (Fig. 5C,F) indicating detrimental effects of OPPM exposure on the fertilizing ability of the buffalo spermatozoa.

## Discussion

**Figure 6:**
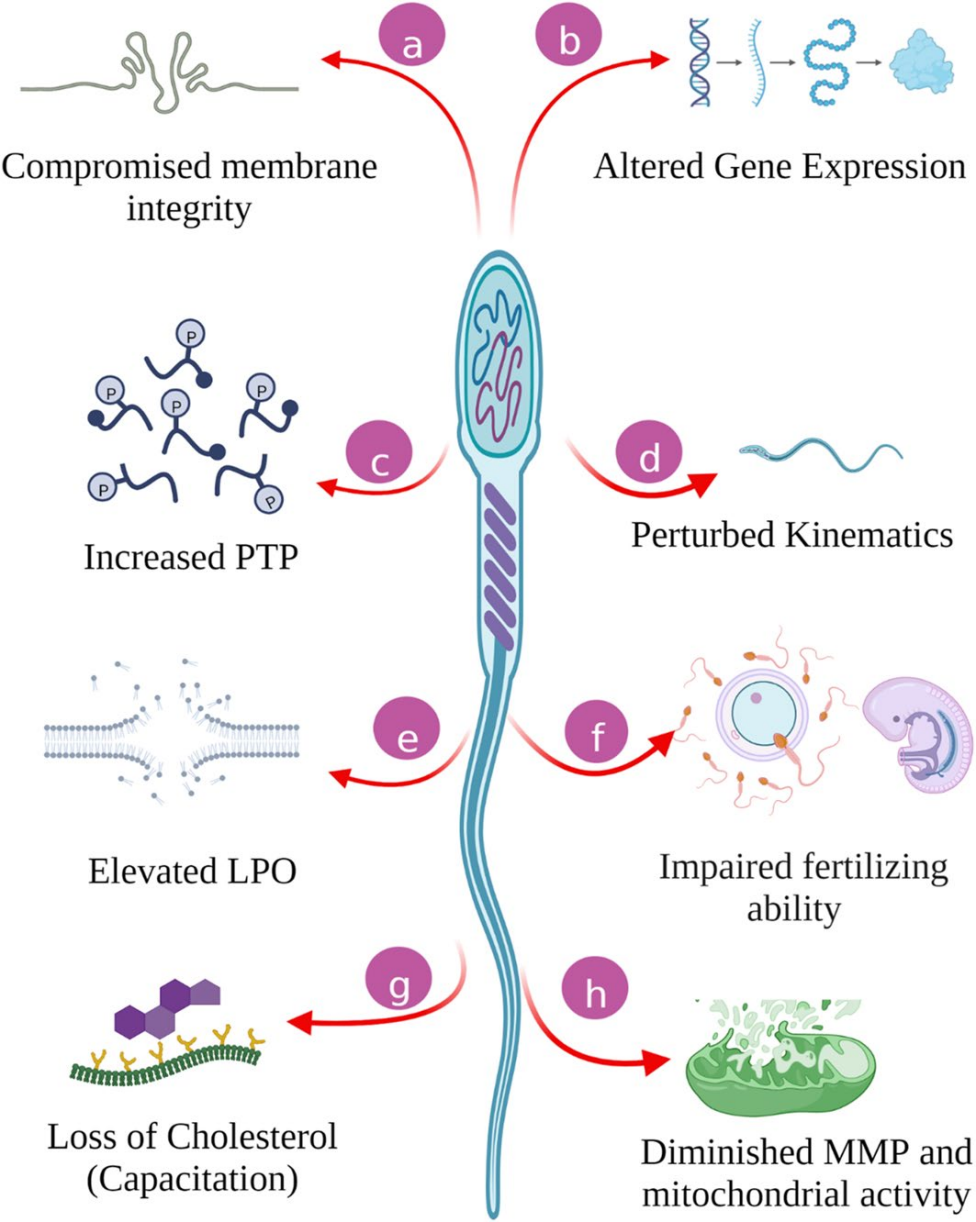
The overall effect of low-dose acute exposure of OPPMs on buffalo spermatozoa. The buffalo sperm were incubated with 0.5, 1, and 2 μM of Omethoate, Paraoxon methyl, TCPy for 2 h which compromised the structural and functional integrity of the exposed sperm as discussed in the text. *PTP* protein tyrosine phosphorylation, *LPO* lipid peroxidation, *MMP* mitochondria membrane potential.

This study was undertaken to assess the effects of acute exposure to three OPPMs viz. Omethoate Paraoxon-methyl, and TCPy on the structural and functional integrity of the bubaline spermatozoa. The OPPs and their metabolites (e.g. oxons) are known to persist in the environment, interact with various organisms and pervade long after the withdrawal of exposure. This not only affects the metabolic, physiologic and reproductive health of the exposed parent but also causes pernicious effects in the forthcoming generations. Surprisingly, most of the reproductive toxicological studies on OPPs/OPPMs have focused either on their roles as endocrine-disrupting chemicals (EDCs) or the epigenetic changes induced upon their exposure. The effects of low-dose, acute exposure to OPPMs on mammalian male gamete have not been studied in detail. Our results indicated that acute exposure to OPP metabolites viz. Omethoate, Paraoxon-methyl, and TCPy compromised bubaline spermatozoa function in a dose-dependent manner. The OPPM exposure resulted in impairment of sperm structural and functional integrity, perturbation in kinematic parameters, altered gene expression and decreased fertilizing ability indicating the toxicological implications of acute, low-dose pesticide metabolite exposure (Fig. 6).

Exposure to pesticides and their metabolites (OPPMs) are known to induce deleterious (cytotoxic) effects on the reproductive system of animals through distinct physiological mechanisms, components and pathways and are thus associated with deteriorated sperm quality^38,52–55^. For example, acute exposure (1–3 h) to Roundup (and its major component Glyphosate) has recently been reported to negatively impact the male gametes causing a reduction in sperm viability, mitochondrial activity, motility and acrosome integrity in a dose-dependent manner^55^. Notably, most of the studies on pesticide exposure have reported adverse impacts on one or more SFPs e.g., membrane integrity, MMP, capacitation and motility^54,57,58^. Many of these parameters are correlated with fertility and have been proposed as fertility biomarkers in addition to their reported associations with the life expectancy of the offspring^59–62^. We also observed a rise in the percentage of spermatozoa with impaired SFPs upon exposure to OPPMs (Figs. 1 and 6), particularly at higher doses (≥ 5 μM). The presence of pesticide residues or their metabolites (OPPMs) in the fluids surrounding spermatozoa exerts negative influences on the spermatozoa function (impairing survival and fertility) and is also implicated in developmental malformations or defects in the fetus/offspring^39,54,63,64^. Several studies across multiple mammalian species have focused on the effects and putative mechanisms of action of (organophosphorus) pesticides on the male reproductive system^7,41,57,65^. For instance, both in vivo (via oral gavage) and in vitro exposure to Chlorpyrifos have been shown to interfere with male reproductive functions leading to reduced fertility in mammals^3,66^. Our results agree with the aforementioned studies that exposure to these xenobiotics potentiates the perturbation of functional parameters of mammalian spermatozoa e.g., mitochondrial function and activity (Fig. 1G–I). The oxidative stress associated with pesticide exposure is known to trigger mitochondrial deficiency among other cytotoxic and genotoxic effects^67,68^. The alteration in the mitochondrial activity and function results in the manifestation of oxidative stress thus creating a vicious cycle and is itself entwined in it (discussed below). The buffalo spermatozoa have been reported to contain an elevated amount of unsaturated fatty acids (PUFA) vis-à-vis other bovids, which renders them vulnerable to oxidative stress-induced damage^26–29,69^. We observed a reduced MMP (Fig. 1G–I) along with a concomitant rise in lipid peroxidation (LPO) of bubaline spermatozoa (Fig. 1J–L) which has been demonstrated to be highly associated with Murrah buffalo bull fertility^70^. The exposure of mammalian spermatozoa to OPPs/OPPMs e.g., Methyl parathion or its metabolite, Methyl paraoxon (one of the most potent insecticides) reportedly caused oxidative stress through elevated LPO, as assessed by malondialdehyde production at 7 and 28 days post-treatment (dpt) in male mice^31^. A different OPP, Dimethoate (along with Chlorpyrifos) is amongst the most frequently used OPPs, especially in developing economies, as mentioned previously^3,71^. As expected, the exposure to Dimethoate (through oral gavage) has been reported to affect the reproductive performance of male mice leading to a decline in sperm motility, the fraction of live spermatozoa, hormone levels and alterations (degenerative changes) in testicular histology^19,20^. The Chlorpyrifos metabolite, TCPy and the Dimethoate metabolite, Omethoate are reportedly more toxic than their parent pesticides^13,18,21,22^. Overall, acute exposure to OPPMs negatively impacted the SFPs which are considered biomarkers of mammalian sperm fertility.

It is worth mentioning that capacitation was the only functional parameter that was affected at low OPPM exposure (2 µM, P < 0.01) to buffalo sperm (Fig. 1D–F). Neither the sperm mass motility nor any other SFP was significantly altered at 2 µM OPPM exposure (Supplementary Fig. 1). It has often been observed that such subclinical effects may appear to be subtle but can nonetheless, cause chronic illness through functional alterations in diverse biological processes^50^. The considerable rise in precociously capacitated sperm at low OPPM (Paraoxon Methyl and TCPy) exposure (2 µM) intrigued us to assess similar, capacitation-associated functional alterations e.g., PTP status and buffalo sperm kinematics. The acute exposure of OPPMs induced protein tyrosine phosphorylation (PTP) in the buffalo spermatozoa even at low doses (1 µM). PTP is an important intracellular mechanism that is implicated in many cellular processes e.g., regulating sperm functions. A rise in the tyrosine protein phosphorylation is considered a robust indicator of capacitation, in several species including humans^72^, rodents^73^, pigs^74^, and bovids including buffalo^75,76^. Most OPPs are well-known phosphorylating agents and our results indicate that the same is true for their metabolites (OPPMs) that induced dose-dependent tyrosine phosphorylation in buffalo spermatozoa (Fig. 2). Our observations are in agreement with these studies and are underpinned by the observed rise in the number of precociously capacitated spermatozoa^51,72–76^. Nevertheless, whether this rise in phosphorylation was genotoxic was not assessed in this study.

Exposure of mammalian spermatozoa to the OPPs has also been demonstrated to perturb many of the sperm kinematic parameters e.g., chlorpyrifos exposure to buffalo spermatozoa reportedly results in a reduction in straight line velocity (STR) and average path velocity (VAP)^37^. Most of these parameters are not perceptible by the naked eye, however, exhibit sensitivity to various reproductive toxicants such as OPPs^77^. Many of the velocity and motion characteristics of the buffalo sperm were found to be altered upon OPPM exposure, apparently in response to increased capacitation (Figs. 4 and 1D–F). Many of these characteristics are crucial to the fertilizing ability of the mammalian spermatozoa, for instance, the straight line velocity (STR) has been proposed to play crucial roles in sperm transport through the FRT and oocyte-penetration^77^. Largely, the results of the CASA experiments indicated perturbed motility and velocity parameters upon OPPM exposure that can potentially affect their reproductive function akin to the OPPs^35,38^. Interestingly, the observed alterations in motility-related spermatozoal kinematics parameters have been ascribed to the elevated mitochondrial gene activity affecting ATP production, which results in precocious capacitation^77,78^. The motility patterns, which are very crucial for the fertilizing ability of mammalian spermatozoa are dependent on energy sources^79,80^. Therefore, we selected a panel of mitochondrial metabolic genes to assess if exposure to OPPMs affects mitochondrial activity. We observed an elevated expression of genes involved in mitochondrial bioenergetics upon acute OPPM exposure (Fig. 3). Mitochondrial oxidative phosphorylation (OXPHOS) and glycolysis are the two main pathways to generate ATP^79,80^ and metabolic mitochondrial genes such as ATP6, ATP8 and the NADPH dehydrogenase subunits (ND1, ND2, and ND4) are involved in ATP formation^81,82^. The induction of the OXPHOS pathway is known to produce a large amount of ATP in sperm that in turn induces sperm capacitation and acrosome reaction, as observed in this study^79–84^. As mentioned earlier, the altered mitochondrial function and impaired structural, functional and motility parameters are expected to cause a considerable decline in the fertilizing ability of the spermatozoa e.g. in IVF^83,84^. In agreement with these studies, we observed a significant reduction in the in vitro fertilizing ability of spermatozoa upon low OPPM exposure (0.5 μM, TCPy) (Fig. 5). A rise in the precocious capacitation upon exposure to OPPMs could be implicated in their impaired fertilizing ability since it was the only SFP affected at dosage ≤ 5 μM^82^, Furthermore, it is worth mentioning that the paternal seminal fluid is capable of influencing the developmental programming effects in the progeny and dictating changes in the uterine luminal components^85^. Such exposures can lead to the impairment of various biochemical pathways and high ROS production resulting in low-quality embryos that consequently leads to poor clinical outcomes in IVF programs^37,84,86,87^. A growing body of evidence indicates that prenatal exposure to environmental xenobiotics e.g., OPPMs can adversely affect fertility, in utero and post-natal development and may have multigenerational effects as addressed under the novel, E-DOHaD (Environmentally-induced Developmental Origins of Health and Disease) model of trans- or inter-generational inheritance^17,38,88,89^. For instance, men exposed to pesticide residues had children with an elevated risk of male reproductive developmental disorders including birth defects such as cryptorchidism, hypospadias, reduced fertility, and stunted growth and development leading to clinical manifestations^45,90–92^. Hence, it would be interesting to assess the effects of OPPM exposure at different embryonic cell stages and post-natal stages in the offspring through adulthood.

There are certain caveats and limitations of this study that are worth discussing. For example, the epigenetic changes and genotoxicity of the OPPM exposure were not ascertained. Besides, a study is warranted to understand the effects of cumulative, minimal dosage, acute and chronic exposure on male reproductive physiology. This is because the mechanisms underlying chronic and acute exposure have been proposed to be distinct. For example, in rodents, the mechanisms behind the reduction of fertilizing ability of spermatozoa at 7- and 28-days post-treatment (dpt) of methyl parathion have been ascribed to the sperm surface remodelling events and acrosomal defects, respectively^31,84^. We had chosen OPPM dosage based on previous reproductive toxicology studies wherein a huge variation in the selected concentrations (0.005 μg/mL to 750 μM) was observed^37,38,41^. Importantly, the assessment of pesticide concentration in representative semen samples (as reported in food products and air samples) would additionally help employ the bio-available equivalent doses for similar studies^92–99^. Overall, the spermatotoxic effects of three OPPMs used in this study appear to share the mechanisms of toxicity as reported for various their parental OPPs^100,101^. The dose-dependent rise in PTP, LPO, and OXPHOS gene expression indicated the involvement of regulatory protein modifications (phosphorylation) and mitochondrial function in mediating the reproductive toxicity (decreased fertility) of OPPMs.

## Conclusion

Exposure to environmental pollutants such as OPPs and their metabolites (OPPMs) appears to be one of the preponderant, previously unidentified pathological factors to be associated with idiopathic male infertility^102,103^. By using the mammalian (Bubaline) spermatozoa as a model, our results revealed that the transient exposure to OPPMs viz. Paraoxon-methyl, Pmethoate and TCPy at low doses (0.5–2 μM) detrimentally affects the functions of the male gamete. Acute exposure to OPPMs resulted in elevated membrane damage, perturbed mitochondrial function, and increased phosphorylation leading to precocious capacitation and impaired fertility. Nevertheless, caution should be exercised while extrapolating the results of these in vitro models to in vivo studies. Moreover, since most couples often share lifestyle habits and diet choices, occupy the same niche, and together transmit the molecular memory of their past environmental experience to their offspring (equal genetic contribution) both parents should be considered in future reproductive toxicity studies. Notably, a male exposed to pesticides (bodily fluids) may also render a female’s uterine environment susceptible to exposure during coitus^104,105^.

## Methods

Reagents All chemicals, media and reagents including the commercial formulation of OPPMs used in this study were procured from Sigma-Aldrich Chemical Co. Ltd, (USA) unless stated otherwise. All plasticware was procured from Nunc Inc. (ThermoScientific, USA). The OPPM formulations were diluted with dimethyl sulfoxide (DMSO).

### Sample collection and processing

Frozen semen straws of Murrah buffalo bulls (N = 9) were procured from the Artificial Breeding Research Centre (ABRC), ICAR-NDRI, India. The straws were thawed by immersing them in a water bath at 38 °C for 30 s. The contents were collected into 15 mL centrifugation tubes containing 2 mL working Sp-TALP medium (1 mM 60% Na-lactate and 0.98 mM Na-Pyruvate in 2X filtered stock mixed with an equal amount of Mili-Q water) (Supplementary sheet—Methods: Table 1). The spermatozoa were separated from the seminal plasma and extender components by centrifuging at 280×*g* for 6 min (thrice) in the working Sp-TALP medium. The supernatant was discarded each time and the sperm pellet obtained after the final wash was subject to the swim-up technique. The obtained fraction containing the motile spermatozoa was suspended in 250 μL working-NCM (non-capacitating, Sp-TALP medium). Since the semen straws were procured from a commercial government-funded farm that operates under standard conditions, specific authorization from the Ethics Committee was not required to conduct this study.

### Experimental design

The motile spermatozoa (10 × 10^6^) obtained from the previous step were incubated with different concentrations (0.5 µM, 1 µM, 2 µM, 5 µM, 10 µM, 20 µM) of three pesticide metabolites of the three most rampantly used OPPs viz. Omethoate, Paraoxon-methyl and 3,5,6-Trichloropyridinol or TCPy^106^ for 2 h at 37 °C in a CO_2_ incubator along with negative control (no pesticide metabolites) and vehicle control (DMSO only). The continuum of doses selected for this study was based on the previously reported concentrations employed in various in vitro and in vivo reproductive toxicological studies on mammalian spermatozoa survival and function^37,39,41,107^. Likewise, the time of incubation was decided based on previously published literature on acute toxicity, most of which have reported an incubation time between 1 and 3 h^37,41,56^.

### Sperm function parameters (SFPs)

After incubation with OPPMs, the control and treated buffalo spermatozoa were evaluated for intactness of structural and functional integrity. The examination of sperm-membrane integrity, acrosome reaction and lipid peroxidation were done by using carboxyfluorescein diacetate-propidium iodide (CFDA-PI) and 4, 4-Difluoro-4-bora-3a, 4a-diaza-s-indacene (BODIPY) dyes, respectively, as per the method described by Singh and colleagues^69^. However, the assessment of mitochondrial membrane potential (MMP) by JC-1 (5,5′,6,6′-tetrachloro-1,1′,3,3′-tetraethylbenzimidazolyl-carbocyanine iodide) and capacitation status by Chlortetracycline (CTC), a fluorescent chelate probe of Ca^2+^ was done by the methods described by Saraf and co-workers^108^. Thereafter, the excess stains were removed by washing the stain-incubated spermatozoa with 200 µL of sperm-TALP by centrifugation at 800×*g* for 3 min. The pelleted spermatozoa were used to make a thin smear onto which a few drops of mounting medium, Dabco® 33-LV were placed and was observed at 1000 × magnification under an Olympus BX-51 fluorescence microscope using appropriate filters. A minimum of n = 200 spermatozoa (in triplicates) were evaluated, in a minimum of 10 fields for observing fluorescent patterns. The images of the two filters were merged to obtain the final image, wherever required.

### Protein tyrosine phosphorylation (PTP) status

The protein tyrosine phosphorylation (PTP) status of the buffalo spermatozoa was assessed using an indirect immunofluorescence assay as described by Saraf et al.^109^. The higher doses of ≥ 10 µM of the OPPMs were omitted from further experiments (explained later). The control and the treated spermatozoa were washed with PBS at 300×*g* for 5 min and a 20 µL sperm suspension was then smeared onto a clean glass slide and air dried. Thereafter, the spermatozoa were fixed in 4% paraformaldehyde for 1 h at 4 °C and washed with PBS (3 ×). The spermatozoa were permeabilized using methanol and the slides were blocked using 5% BSA in PBS for 2 h at room temperature. Subsequently, the smears were incubated with monoclonal anti-phosphotyrosine antibody (P1869; Sigma, 1:100) in 1% BSA for 3 h at 37 °C and then washed with PBS (3 ×). Afterwards, the smears were incubated with FITC-conjugated anti-mouse IgG antibody produced in goat (F4018; Sigma, 1:100) and then washed thoroughly with PBS. After the final washing step, the coverslip was mounted onto a dried glass slide. A drop of mounting medium, Dabco® 33-LVwas placed on the slide and the cells were then observed under a BX-51 Olympus fluorescence microscope at 1000 × magnification using a FITC filter. The spermatozoa were assessed for the percentage of different phosphorylation patterns and a minimum of 200 spermatozoa were counted (in three technical replicates) across the slide. The three patterns of tyrosine phosphorylation (pattern NP—no fluorescence; pattern EM—fluorescence over the equatorial region and mid-piece and pattern AEM—fluorescence over the acrosomal area, equatorial region and mid-piece) were counted and expressed as percentages.

### Expression dynamics of the metabolic and oxidative stress-related genes

Total RNA was isolated from different experimental groups and the control group of spermatozoa as described previously by Batra and colleagues^110^ using the TRI Reagent, RNA isolation reagent (Sigma-Aldrich, USA). The isolated RNA was quantified using a Nanodrop ND-1000 UV–Vis spectrophotometer (NanoDrop Technologies Inc., Wilmington, DE, USA). The cDNA synthesis and RT-qPCR optimization were done as described by Batra et al.^110^ (Supplementary sheet-Methods). The primer designing tool at NCBI, the Primer-BLAST was used to design primers for the metabolic genes (ATP 6, ATP 8, COX 2, CYT B, ND1 and ND2) and two reference genes viz. GAPDH (glyceraldehyde-3-phosphate dehydrogenase) and β-actin in the buffalo spermatozoa. Intron-spanning primers were designed, wherever possible and the self-annealing sites, mismatches and secondary structures in the primers were checked using OligoCalc^111^. The specificity of each set of primers was again checked using the BLAST alignment tool and in silico PCR^112^ was run for each set of primers before commercial synthesis (Sigma-Aldrich, USA). The MIQE^113^ guidelines were followed at every step, wherever possible. The relative quantification of all the genes was done on a Bio-rad CFX-96 Touch Deep Well Real-Time PCR system platform using the iTaqUniversal SYBR Green Supermix (Bio-Rad, USA) in a 10 μL reaction mix. The thermal profile was 95 °C for 5 min, 40 cycles consisting of denaturation at 95 °C for 15 s, annealing at variable optimized temperatures for 20 s, extension at 72 °C for 20 s, followed by the melt curve protocol with 10 s at 95 °C and then 60 s each at 0.5 °C increments between 65 and 95 °C. The melt curve analysis ensures a specific, unique product formation and ascertains primer dimer formation. A no-template control (NTC) was included in each plate to confirm the absence of nucleic acid contamination. The mean sample C_q_ (Cycle of quantification) values for the various metabolic and oxidative stress-related genes were calculated for duplicate samples and their relative expression was calculated by ΔΔCt method, as described previously^114^.

### Sperm kinematics using computer-assisted sperm analyzer

The spermatozoa incubated with pesticide metabolites (OPPMs) were subject to computer-assisted sperm analysis (CASA) for estimating the velocity and motion parameters of the buffalo spermatozoa. Computer-assisted sperm analyzer (IVOS12.1, Hamilton-Thorne Biosciences, Beverly, MA, USA) was used to evaluate the kinetic characteristics. The motility and movement parameters like the curvilinear velocity (VCL, µm/s), linear velocity (VSL, µm/s), average path velocity (VAP, µm/s), the mean amplitude of lateral head displacement (ALH, µm), the percentage of linearity i.e. the ratio between VSL and VCL (LIN, %), the straightness coefficient which is the ratio between VSL and VAP (STR, %) and the frequency with which the actual sperm trajectory crossed the average path trajectory (BCF, Hz) were recorded in duplicates for all the experimental groups. The CASA software settings were as follows: temperature = 38 °C, frame rate = 60 Hz, frames acquired = 30, minimum contrast = 35, minimum cell size = 5 pixels, cell size = 9 pixels, cell intensity = 110 pixels, progressive cells (VAP cut-off = 50 m/s, STR cut-off = 70%), slow cells (VAP cut-off = 30/s and VSL cut-off = 15/s). The spermatozoa (N = 500) were observed in a minimum of five optical fields around the central reticulum of the chamber for sperm motility analysis.

### IVF (in vitro fertilization) Study

The IVF experiments were done as previously explained by Batra et al.^105^. Briefly, buffalo ovaries from the slaughtered animals were transported to the laboratory in physiological saline (0.9%, w/v NaCl) containing strepto-penicillin (50 mg/L) within 2–3 h of slaughter. After washing the ovaries with normal saline, the follicular fluid was aspirated using a vacuum pump (Cook) in HEPES-buffered hamster embryo culture (HH) medium. After extensive washing with HH medium, 15 cumulus-oocyte complexes (COCs) were placed in 100 µL droplets of maturation medium (HEPES buffered TCM199 modified with 10% (v/v) fetal bovine serum, (FBS), 0.005% (w/v) streptomycin, 0.01% (w/v) sodium pyruvate and 0.005% (w/v) glutamine supplemented with 5.0 μg/mL FSH, 10 μg/mL LH, 1 μg/mL estradiol 17-β, 50 ng/mL epidermal growth factor (EGF), 64 μg/mL cysteamine and 50 μL ITS). The dishes were cultured in duplicate for 24 h at 38.5 °C in a humidified atmosphere of 5% CO_2_ in an incubator. The IVF (in vitro fertilization) was carried out using 50 µL of spermatozoa suspension (1 × 10^6^/mL) from OPPM-treated samples along with a control (vehicle). In vitro fertilization (IVF) also was carried out as per the procedure described in the abovementioned study. The IVC (in vitro culture) droplets of 100 µL were prepared following the progressive removal of the media from IVM droplets which was later replaced with IVC medium (mCR2aa—modified Charles Rosenkrans medium with amino acids containing 0.8% BSA, fatty acid-free, and 50 mg/mL gentamicin). After 12 h of IVF, oocytes were denuded mechanically by brief vortexing. The oocytes were subsequently washed five times in the IVC medium and transferred to IVC droplets in groups of 15. A total of 50 µL of IVC medium was replaced with fresh medium after 48 h. The replacement medium constituted of mCR2aa supplemented with 10% (v/v) FBS, 50 mg/mL of gentamicin and 0.8% BSA (fatty acid-free). The second replacement was done 48 h after the first replacement. The cleavage rates were assessed at the time of the first medium replacement after IVC while blastocyst rates were determined 7 days post-IVC.

### Statistical analysis

The differences in the various SFPs, heterogeneity in phosphorylation, differential gene expression levels and kinematic parameters and the differences in cleavage and blastocyst rates within the different dosage groups were subjected to the Shapiro–Wilk normality test while the Brown–Forsythe test was employed to test the differences in their variance (standard deviation). One-way ANOVA and Tukey’s post-hoc test were used to measure the differences in the function parameters of the spermatozoa, phosphorylation status, mitochondrial gene expression, kinematic behaviour and the cleavage and blastocyst formation rates between the different dosage groups on GraphPad Prism 8.0 (for Windows, GraphPad Software, La Jolla California USA, www.graphpad.com). A P-value < 0.05 was considered to be statistically significant.

## Supplementary Information

Supplementary Information 1.

Supplementary Legends.

Supplementary Figure 1.

Supplementary Figure 2.

Supplementary Figure 3.

Supplementary Figure 4.

## Data Availability

All data generated or analyzed during this study are included in this published article and its supplementary information files. Supplementary Material & Information: Supplementary sheet-Methods and Supplementary Figs. 1, 2, and 3.
