# Supplementary material for: Acute exposure to organophosphorus pesticide metabolites compromises buffalo sperm function and impairs fertility
Source: Sci Rep. 2023 Jun 5;13:9102. doi: 10.1038/s41598-023-35541-6 (PMC10241957; doi:10.1038/s41598-023-35541-6)
Supplement: Supplementary file 1 — Supplementary Information 1. [file 41598_2023_35541_MOESM1_ESM.docx]

**Supplementary Methods-Table 1: Composition of non-capacitating TALP media (NCM)-2x stock**

| **Component** | **Concentration** |
| --- | --- |
| **NaCl** | 100 mM |
| **HEPES** | 10 mM |
| **KCl** | 3.1 mM |
| **EDTA** | 0.4 mM |
| **MgCl_2_.6H_2_O** | 0.4 mM |
| **NaH_2_PO_4_.2H_2_O** | 0.3 mM |

**RT-qPCR optimization**

a)Annealing temperature and cDNA integrity:

The annealing temperatures of all the primers were optimized by the conventional gradient PCR (Biometra, Analytik Jena, Germany). The annealing temperature at which the brightest and sharpest band was obtained was used for proceeding further. Briefly, Nuclease-free water, dNTPs, forward and reverse primers (1µM), Taq DNA polymease, PCR buffer were added in a 25µl reaction volume. The reaction was carried out under following conditions: Initial denaturation at 92^o^C for 3 minutes, denaturation at 92^o^C for 45 seconds, annealing-variable for 40 seconds, extension at 72^o^C for 40 seconds and a final extension for 10 minutes at 72^o^C.

b) RT-qPCRand reaction efficiency

A twofold dilution series was used to calculate the efficiency of qRT-PCR reaction for each primer for all the 5 MRT segments. The standard curves were prepared as the semi-log regression line plot of Cq value versus the log of the relative cDNA concentrations used in the dilution series. The primers yielding slopes between -3.17 and -3.0, in the semi-log regression line plots, with efficiencies corresponding to 106% and 115% respectively and R^2^ ≥ 0.98 were used for RT-qPCR. Real-time PCR efficiencies were calculated, according to E = 10[–1/slope] (A). If these criteria were not meant the reactions were either repeated or the primer was re-synthesized.

c) Validation of reference genes

The reference genes were chosen based on available literature (B) and genevestigator expression database and meta-analysis system using refGenes application (C) using *Bos taurus* as reference. For accurate normalization of the RT-qPCR data, geometric averaging of the two reference genes viz. GAPDH and β-actin were used. Normalizingthe gene expression to multiple reference genes in order to give a more reliable baseline for the calculation of relative gene expression using qRT-PCR is common practice (D)

1. Svec, D., Tichopad, A., Novosadova, V., Pfaffl, M. W., &Kubista, M**., How good is a PCR efficiency estimate: Recommendations for precise and robust qPCR efficiency assessments. Biomolecular Detection and Quantification, 3, 9–16, 2015**
2. T. J. Hall, C. McQuillan, E. K. Finlay, C. O’Farrelly, S. Fair and K. G. Meade, **Comparative genomic identification and validation of β-defensin genes in the Ovisaries genome, BMC genomics,18:278, 2017**
3. Hruz T, Wyss M, Docquier M, Pfaffl MW, Masanetz S, Borghi L, Verbrugghe P, Kalaydjieva L, Bleuler S, Laule O, Descombes P, Gruissem W, Zimmermann P**, RefGenes: identification of reliable and condition specific reference genes for RT-qPCR data normalization.. BMC Genomics. 2011 Mar 21;12(1):156.**
4. Jo Vandesompele, Katleen de preter, FilipPattyn, Bruce Poppe, Nadine Van Roy, Anne De Paepe and Frank speleman, **Accurate normalization of real time quantitative RT-PCR data by geometric averaging of multiple internal control genes Genome Biology, 3(7): research0034.1-0034.11, 2002**
