# Supplementary material for: Acute exposure to organophosphorus pesticide metabolites compromises buffalo sperm function and impairs fertility
Source: Sci Rep. 2023 Jun 5;13:9102. doi: 10.1038/s41598-023-35541-6 (PMC10241957; doi:10.1038/s41598-023-35541-6)
Supplement: Supplementary file 2 — Supplementary Legends. [file 41598_2023_35541_MOESM2_ESM.docx]

**Supplementary Fig.1 Motility.** Percent motile spermatozoa in control (no OPPM), vehicle control (DMSO only) and buffalo spermatozoa exposed to 0.5, 1, and 2 μM of OPPMs viz. Omethoate (**A**), Paraoxon methyl (**B**), and TCPy (**C**) for 2 hours.

**Supplementary Fig.2 Sperm Function Parameters** Representative photomicrographs of the buffalo spermatozoa labelled with CFDA-PI (**A**) which were examined under fluorescence epi-illumination at magnification 1000X. PI produces deep red colour entering the dead spermatozoa whereas CFDA fluoresces green with live cells. Spermatozoa labelled with CTC (**B**) which fluoresces bright yellow on binding Ca+2 under UV light. Spermatozoa labelled with JC-1 (**C**) fluoresce apple green indicating functionally intact mitochondria (high MMP) while the appearance of yellow fluorescence along mid-piece upon binding of BODIPY is indicative of lipid peroxidation (**D**).

**Supplementary Fig.3 Protein Tyrosine Phosphorylation** Representative photomicrograph of the buffalo spermatozoa labelled with monoclonal anti-phosphotyrosine antibody (P1869; Sigma). The phosphorylated proteins on the spermatozoa produce bright green fluorescence on acrosomal, equatorial and mid-piece (AEM) or the equatorial and mid-piece (EM) regions.

**Supplementary Fig.4** **CASA** The mean amplitude of lateral head displacement (**A-C**) and beat cross frequency (**D-F**) evaluated in the buffalo spermatozoa upon exposure to 0.5, 1, and 2μM of Omethoate (**A, D**), Paraoxon methyl (**B, E**), TCPy (**C, F**) by Computer-assisted sperm analyzer (IVOS12.1, Hamilton-Thorne Biosciences, Beverly, MA, USA).
