# Supplementary figures and images for: Acute exposure to organophosphorus pesticide metabolites compromises buffalo sperm function and impairs fertility
Source: Sci Rep. 2023 Jun 5;13:9102. doi: 10.1038/s41598-023-35541-6 (PMC10241957; doi:10.1038/s41598-023-35541-6)

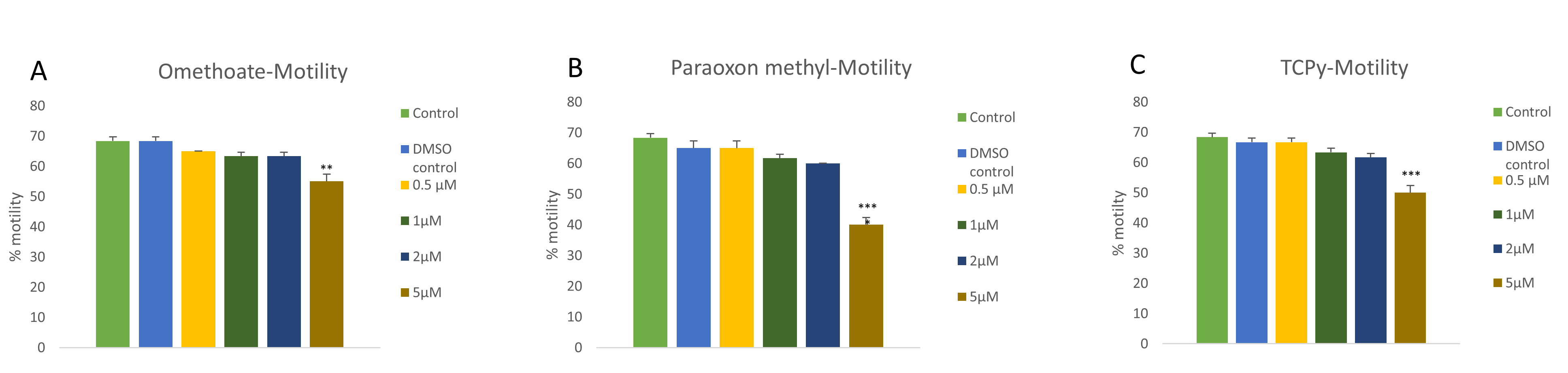

Supplement: Supplementary file 3 — Supplementary Figure 1. [file 41598_2023_35541_MOESM3_ESM.tif]

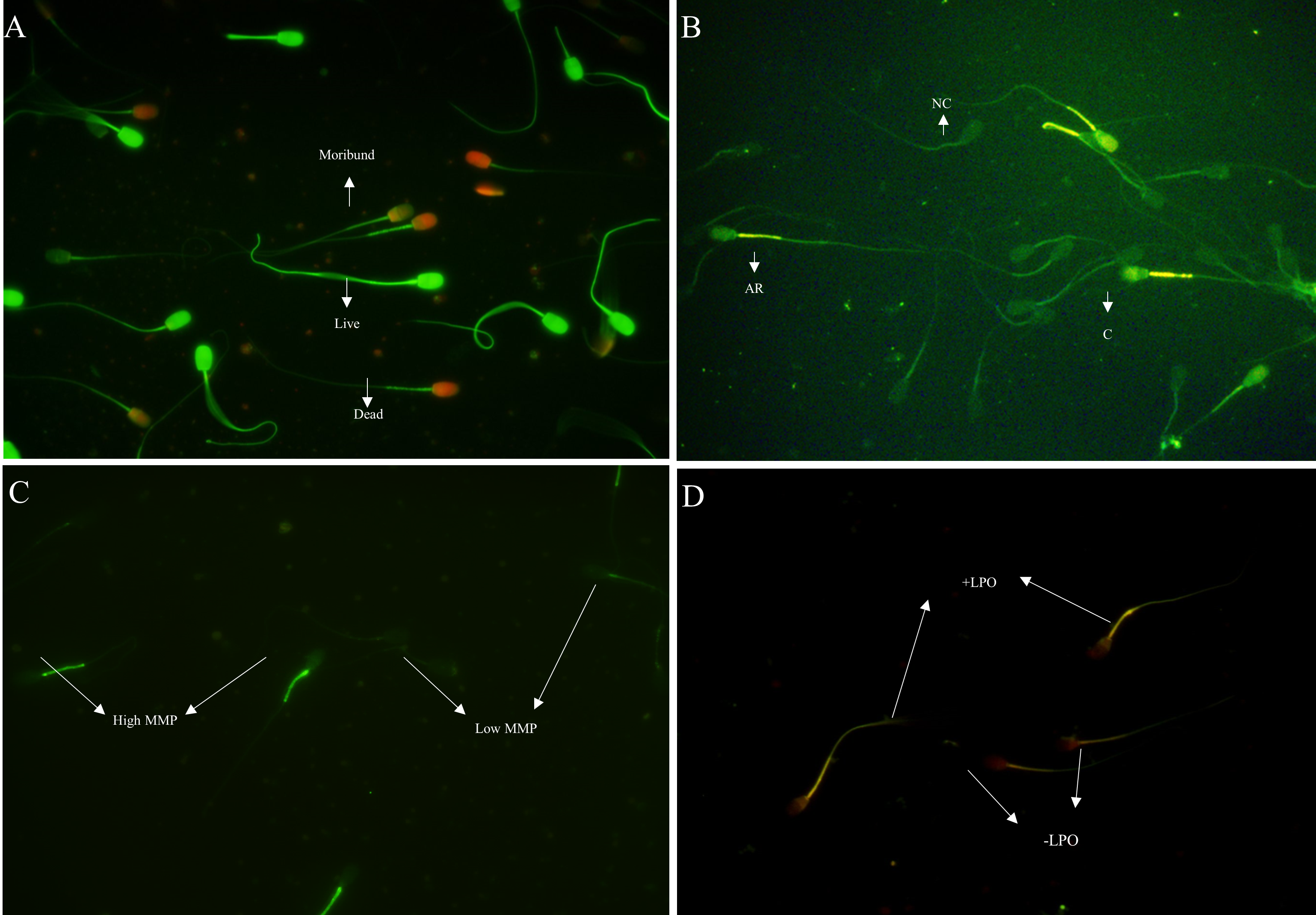

Supplement: Supplementary file 4 — Supplementary Figure 2. [file 41598_2023_35541_MOESM4_ESM.tif]

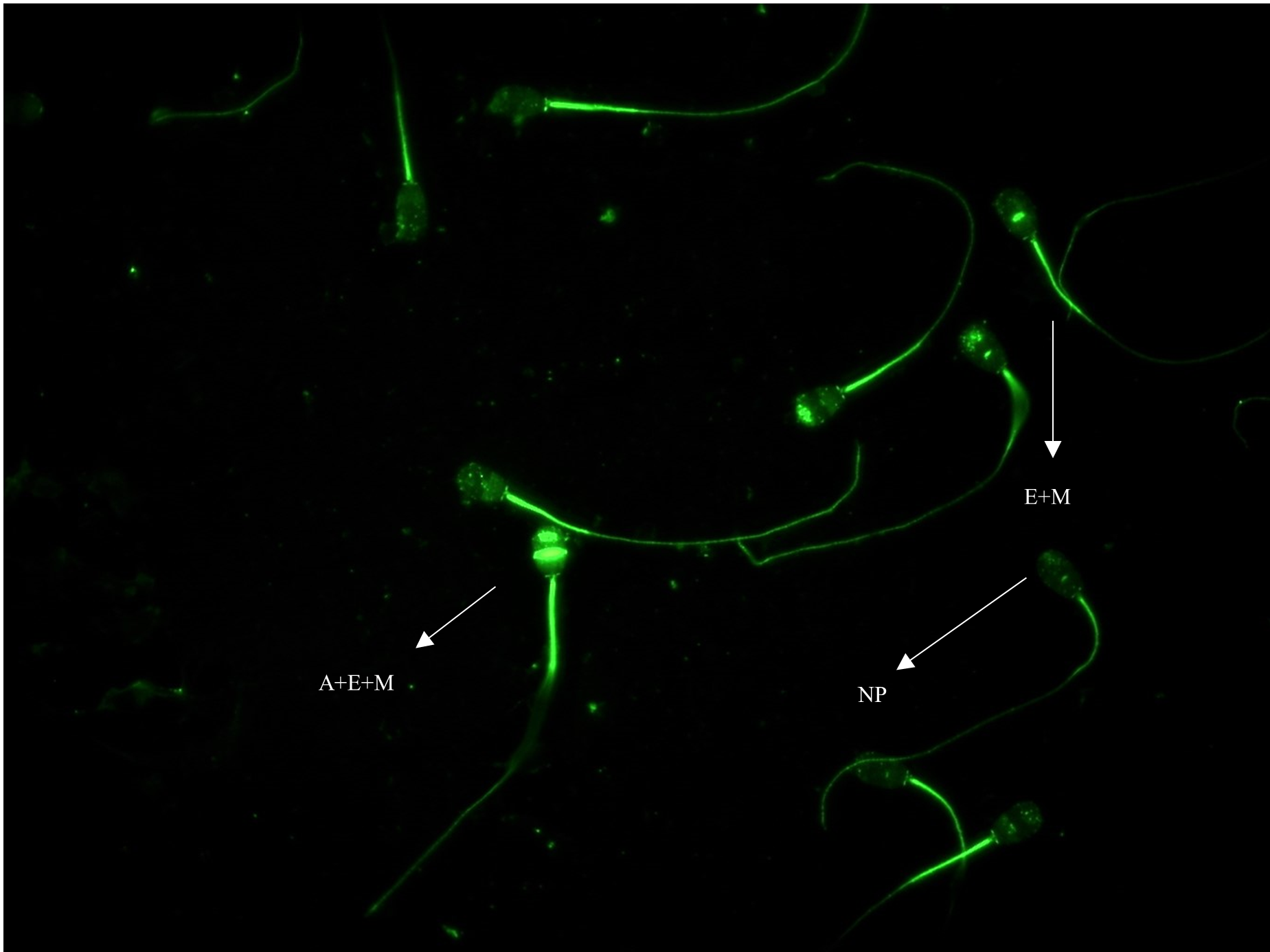

Supplement: Supplementary file 5 — Supplementary Figure 3. [file 41598_2023_35541_MOESM5_ESM.tif]

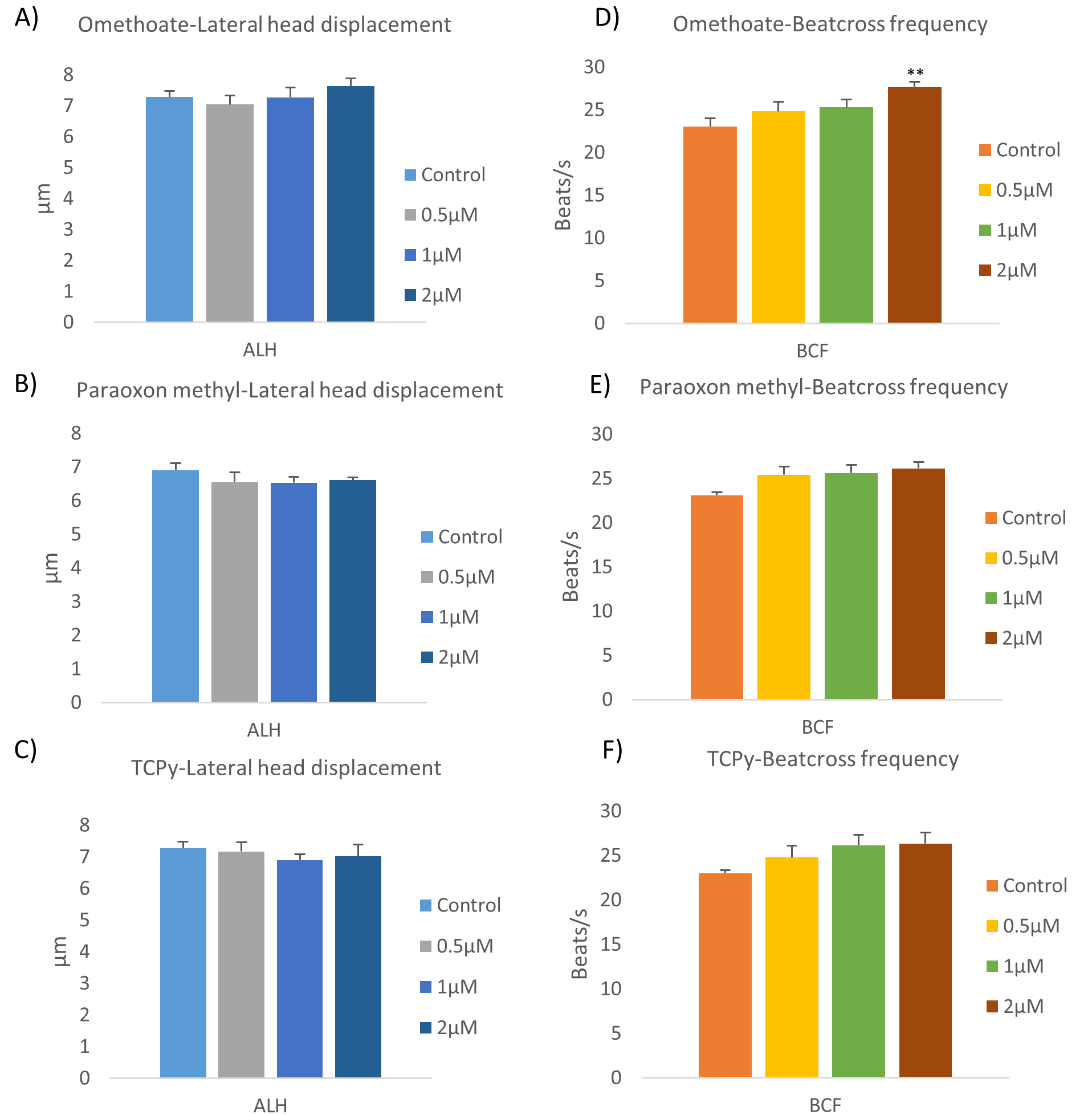

Supplement: Supplementary file 6 — Supplementary Figure 4. [file 41598_2023_35541_MOESM6_ESM.tif]
